# Supplementary material for: Identification of RNA 3´ ends and termination sites in Haloferax volcanii
Source: RNA Biol. 2020 Feb 10;17(5):663–76. doi: 10.1080/15476286.2020.1723328 (PMC7237163; doi:10.1080/15476286.2020.1723328)
Supplement: Supplemental Material [file krnb-17-05-1723328-s001.zip › Supplementary information/SupplementaryData.pdf]

Supplementary Data

Supplementary Data consist of:

A. Supplementary Methods (page 1),
B. Supplementary Figures 1-7 (page 3),
C. Legends to Supplementary Tables 1-4 (page 9),
D. Supplementary Tables 5-11 (page 11),
E. Reference (page 31).

A. Supplementary Methods

Library construction and next generation sequencing

All RNA fractions were treated with RiboZero kit (Epicentre) to remove the ribosomal RNAs. The RNA fraction "+TEX" was first incubated with T4 polynucleotide kinase (to phosphorylate 5´ hydroxyl groups) and subsequently with Terminator exonuclease (TEX) (Epicentre) to remove the RNA species which carry a 5´ mono-phosphate, thus this RNA fractions is enriched in primary transcripts. After the TEX treatment an adenylated oligonucleotide (3´ Illumina sequencing adapter) was ligated to the 3´ OH ends of the RNAs. Then, first-strand cDNA synthesis was performed using M-MLV reverse transcriptase and the 3´ adapter as primer. The first-strand cDNA was fragmented and to the 3´ ends of the cDNA fragments the 5´ Illumina sequencing adapter was ligated. Finally, cDNAs were PCR-amplified using a high fidelity DNA polymerase (Herculase II Fusion DNA Polymerase, Agilent 600679) and TruSeq Dual Index PCR primers.

The RNA fraction "-TEX" was not treated with TEX. An adenylated oligonucleotide (3´ Illumina sequencing adapter) was ligated to the 3´ OH ends of the rRNA depleted RNAs. Then, first-strand cDNA synthesis was performed using M-MLV reverse transcriptase and the 3´ adapter as primer. The first-strand cDNA was fragmented and to the 3´ ends of the cDNA fragments the 5´ Illumina sequencing adapter was ligated. Finally, the cDNA fragments were PCR-amplified using a high fidelity DNA polymerase (Herculase II Fusion DNA Polymerase, Agilent 600679) and TruSeq Dual Index PCR primers.

For preparation of the RNA fraction RNAseq (also termed transcriptome), the RNA was first fragmented using ultrasound (4 pulses of 30 s each at 4°C). Then, an oligonucleotide adapter was ligated to the 3´ end of the RNA molecules. First-strand cDNA synthesis was performed using M-MLV reverse transcriptase and the 3´ adapter as primer. The first-strand cDNA was purified and the 5´ Illumina TruSeq sequencing adapter was ligated to the 3´ end of the antisense cDNA. The resulting cDNA was PCR-amplified to about 10-20 ng/µl using a high fidelity DNA polymerase (Herculase II Fusion DNA Polymerase, Agilent 600679).

For all RNA fractions the cDNAs were purified using the Agencourt AMPure XP kit (Beckman Coulter Genomics) and were analysed by capillary electrophoresis. For Illumina sequencing, the cDNAs were pooled in approximately equimolar amounts. The cDNA pool was eluted in the size range of 200 –500 bp from a preparative agarose gel. An aliquot of the size fractionated cDNA pool was analysed by capillary electrophoresis. The cDNA pool was sequenced on a Illumina HiSeq 2000. The number of obtained reads, mapped and unmapped reads, respectively, is shown in Supplementary Table 6.

Identification of operons

To identify operons in the *H. volcanii* genome, we used Rockhopper (1), a tool that maps prokaryotic RNA sequencing data onto a reference genome. Given an annotation, the output of the program includes a list of operons retrieved from the RNAseq data. We used the latest version of the *Haloferax* genome annotation (2) as a customised input for Rockhopper.

Altogether, we found 632 operons and 2,752 genes that were monocistronic. The majority of operons contained two genes, and the highest number of genes in an operon was 25 (Supplementary Table 9).

Analysis of transcription efficiencies in operons

For each first and each last gene in an operon, we calculated the TPM value based on RNASeq reads. For each operon, we then calculated the ratio of the TPM value of the first gene divided by the TPM value of the last gene. This was done separately for the set of all operons without internal TTS signals and the set of all operons showing internal TTS signals. For reasons of visualization, we created the boxplot based on the logarithmic ratios (Supplementary Figure 7). We see similar mean values for all replicates and conditions, however, there is a relatively high number of outliers on both sides. Outliers on top of the boxes show operons where the TPM value of the first gene is higher than the one of the last gene. Outliers below the box show the opposite, the TPM value of the first gene is lower than the TPM value of the last gene.

To get more information about operons and the corresponding genes, we list 7 operons that occur for all three replicates as outliers on top of the box (high ratio, Supplementary Table 10) and 10 operons that occur in all three replicates as outliers below the box (low ratio, Supplementary Table 11). The tables list general information (rows with blue background) about the operon such as an ID, start and end coordinates, the number of internal TTS, the strand, the number of corresponding genes and the calculated ratios (in normal scale). For each operon, corresponding genes are then listed separately (rows with white background) with gene ID, start and end coordinates, strand, length and annotation.

B. Supplementary Figures 1 - 7

Supplementary Figure 1. 3´ UTR lengths identified with DSM. Histogram showing the length distribution of the identified 3´ UTRs. The median 3´ UTR length was 58 nucleotides. x-axis: 3´ UTR length (distance between TTS and 3´ end of annotated upstream gene), y-axis: frequency of data points falling inside one of the equi-distanced areas of the histogram.

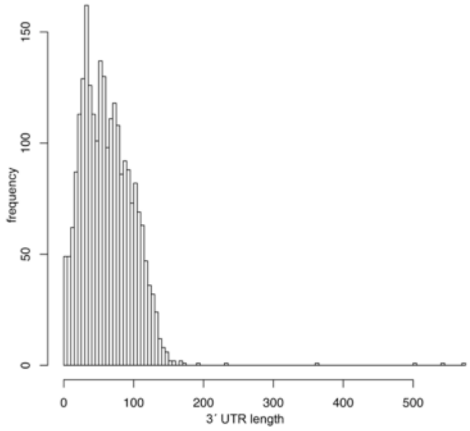

Supplementary Figure 2. Termination site for the *pilA2* gene. The termination site downstream of the *pilA2* gene (HVO_2062) is shown. The termination site is indicated by an orange rectangle in the middle panel. The annotated gene is shown in the upper panel, and the genomic location is indicated below the annotation and above the coverage as a blue line with coordinates given in Mb. The read end counts are shown in the lower panel (read counts per position), and DSM data are reported as binary signals; thus, either a signal is present or not. The Figure was created using the R package Gviz[23].

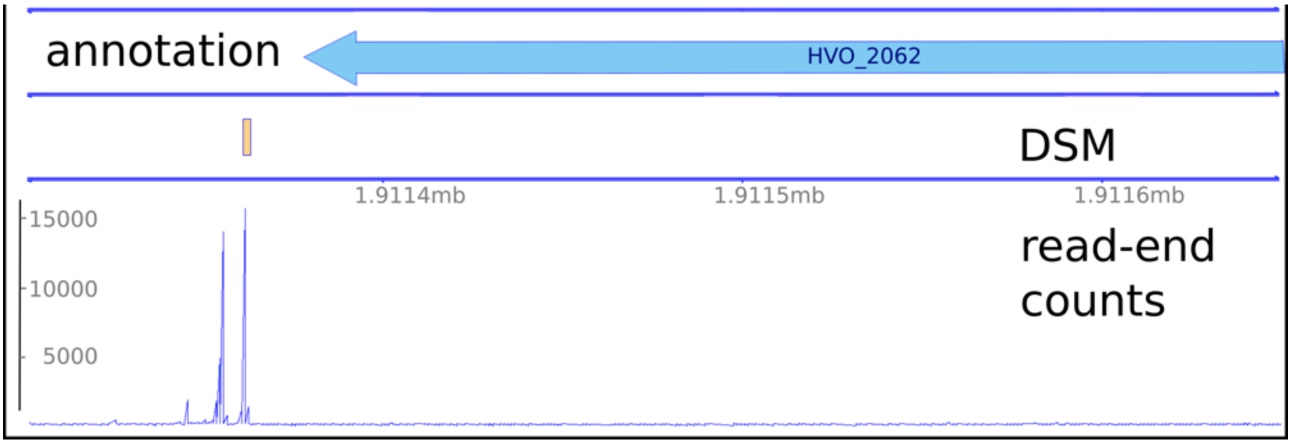

Supplementary Figure 3. Analysis of up- and downstream regions for TT sites identified with DSM. A. Intergenic and B. coding regions were investigated. For the TTS identified with DSM, 45 nucleotides up- and downstream of the termination site (x-axis) were analysed in terms of a) nucleotide enrichment at each position (left y-axis) and b) the hybridisation energy (right y-axis). The colour scheme for the four nucleotides is shown at the upper left, and the energy data are shown with a black line. Hybridisation energies were calculated based on the binding energies between the DNA template and RNA in the area behind the RNA polymerase (for details, see Materials and Methods).
Determination of the hybridisation energy ∆G between the nascent mRNA and the template DNA around the TTS revealed that the hybridisation energy increased at termination sites, which indicates a destabilisation of the DNA-RNA hybrid, supporting transcription termination. This was especially prominent for sites in intergenic regions. Nucleotide enrichment analyses for the intergenic TTS showed a clear preference for thymidine two to seven nucleotides upstream of the TTS. The number of adenosines was slightly higher five to 21 nucleotides downstream of the TTS. The region upstream of the TTS (up to position -8) was depleted of cytidine (with the strongest C depletion at position -3) and guanidine nucleotides (with the strongest G depletion at -1). The common motif for termination, as deduced from the nucleotide enrichment, showed that T frequently occurred directly upstream of the termination site, with C higher than average close to the termination site, A being enriched downstream of the termination site and G being depleted. Within coding regions, T enrichment upstream of the termination site and A enrichment downstream were found. C and G were both depleted around the TTS.

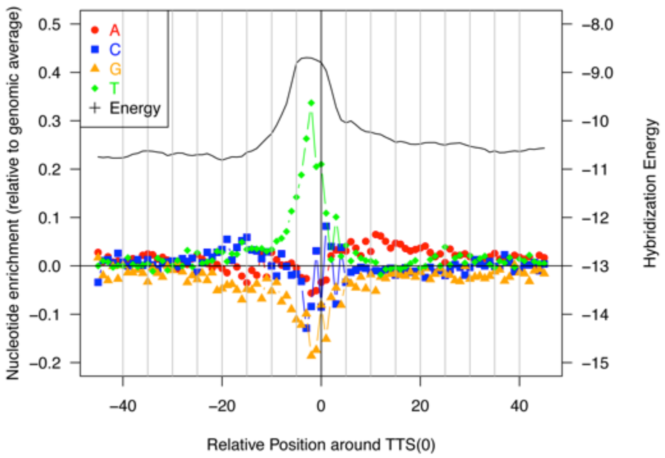

A.

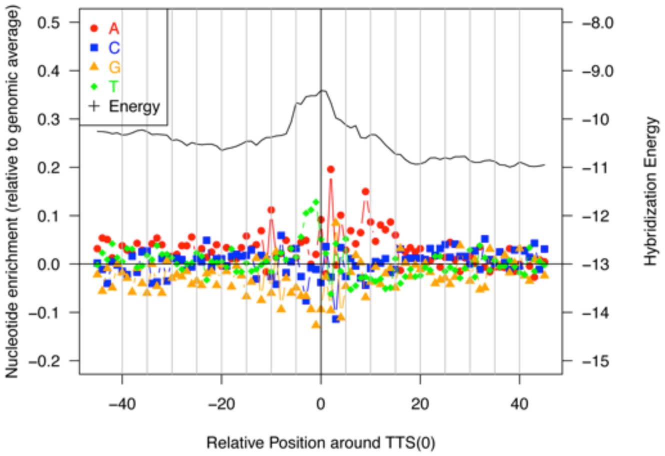

B.

Supplementary Figure 4. Alignment of sequences upstream of the TTS. The alignment is part of the graphclust output and shows the 20 sequences that fit best (compare also Figure 9). Sequence identifiers are internal graph clust identifiers. The TTS is located at position 100, as sequences were cut with 100 nt upstream and 2 nt downstream of the TTS.

```
                   ....((((.(((...(((.(((((((((.(((...)))..))))))))).)))...))).
SEQ6091_14_102_+   -UGAUCCGACGGGCAGCGUGGACGGCCCUCGCGACGCGUCGGGCCGUCCCCGCGAACCGG 59
SEQ5434_14_102_+   -UGAUCCGACGGGCAGCGUGGACGGCCCUCGCGACGCGUCGGGCCGUCCCCGCGAACCGG 59
SEQ5872_14_102_+   -UGAUCCGACGGGCAGCGUGGACGGCCCUCGCGACGCGUCGGGCCGUCCCCGCGAACCGG 59
SEQ5907_11_99_+    -UGAUUCGACGGGCAGCGUGGACGGCCCUCGCGGUGCGUCGGGCCGUCCCCGCGAACCGG 59
SEQ6084_11_99_+    -UGAUUCGACGGGCAGCGUGGACGGCCCUCGCGGUGCGUCGGGCCGUCCCCGCGAACCGG 59
SEQ4618_14_102_+   AUGGUCCGUCGAGCAGCGUGGACGGCCCUCGCGGUGCGUCGGGCCGUCCCCGCGAACCGA 60
SEQ6009_14_102_+   -UGGUCCGUCGAGCAGCGUGGACGGCCCUCGCGGUGCGUCGGGCCGUCCCCGCGAACCGA 59
SEQ5738_3_101_+    ----UCCGUCGAGCAGCGUGGACGGCCCUCGCGGUGCGUCGGGCCGUCCCCGCGAACCGA 56
SEQ5491_1_102_+    -UGGUCCGUCGAGCAGCGUGGACGGCCCUCGCGGUGCGUCGGGCCGUCCCCGCGAACCGA 59
SEQ6008_1_102_+    -UGGUCCGUCGAGCAGCGUGGACGGCCCUCGCGGUGCGUCGGGCCGUCCCCGCGAACCGA 59
SEQ5107_1_102_+    AUGGUCCGUCGAGCAGCGUGGACGGCCCUCGCGGUGCGUCGGGCCGUCCCCGCGAACCGA 60
SEQ6089_1_102_+    AUGAUCCGACGGGCAGCGUGGACGGCCCUCGCGACGCGUCGGGCCGUCCCCGCGAACCGG 60
SEQ5432_1_102_+    AUGAUCCGACGGGCAGCGUGGACGGCCCUCGCGACGCGUCGGGCCGUCCCCGCGAACCGG 60
SEQ5912_1_100_+    ---AUCCGACGGGCAGCGUGGACGGCCCUCGCGACGCGUCGGGCCGUCCCCGCGAACCGG 57
SEQ4561_1_100_+    ---AUCCGACGGGCAGCGUGGACGGCCCUCGCGACGCGUCGGGCCGUCCCCGCGAACCGG 57
SEQ5871_2_101_+    ---AUCCGACGGGCAGCGUGGACGGCCCUCGCGACGCGUCGGGCCGUCCCCGCGAACCGG 57
SEQ4492_1_100_+    ---AUCCGACGGGCAGCGUGGACGGCCCUCGCGACGCGUCGGGCCGUCCCCGCGAACCGG 57
SEQ5613_1_102_+    -UGAUCCGACGGGCAGCGUGGACGGCCCUCGCGACGCGUCGGGCCGUCCCCGCGAACCGG 59
SEQ5787_1_102_+    -UGAUCCGACGGGCAGCGUGGACGGCCCUCGCGACGCGUCGGGCCGUCCCCGCGAACCGG 59
SEQ5538_1_102_+    -UGAUCCGACGGGCAGCGUGGACGGCCCUCGCGACGCGUCGGGCCGUCCCCGCGAACCGG 59
                   .........10........20........30........40........50.........

                   ....))))..((((((...))))))....(((....)))....
SEQ6091_14_102_+   CCACCGGACGACCGGUUCCACCGGUAACUC------------- 89
SEQ5434_14_102_+   CCACCGGACGACCGGUUCCACCGGUAACUC------------- 89
SEQ5872_14_102_+   CCACCGGACGACCGGUUCCACCGGUAACUC------------- 89
SEQ5907_11_99_+    CCACCAGACGACCGGUUUAGCCGGUAACUC------------- 89
SEQ6084_11_99_+    CCACCAGACGACCGGUUUAGCCGGUAACUC------------- 89
SEQ4618_14_102_+   CCACCAGACGACCGGUUCCACCGGUAACU-------------- 89
SEQ6009_14_102_+   CCACCAGACGACCGGUUCCACCGGUAACUC------------- 89
SEQ5738_3_101_+    CCACCAGACGACCGGUUCCACCGGUAACUCCCGACCGGGCUAG 99
SEQ5491_1_102_+    CCACCAGACGACCGGUUCCACCGGUAACUCCCGACCGGGCUAG 102
SEQ6008_1_102_+    CCACCAGACGACCGGUUCCACCGGUAACUCCCGACCGGGCUAG 102
SEQ5107_1_102_+    CCACCAGACGACCGGUUCCACCGGUAACUCCCGACCGGGCUA- 102
SEQ6089_1_102_+    CCACCGGACGACCGGUUCCACCGGUAACUCCCGUCCGGGCUA- 102
SEQ5432_1_102_+    CCACCGGACGACCGGUUCCACCGGUAACUCCCGUCCGGGCUA- 102
SEQ5912_1_100_+    CCACCGGACGACCGGUUCCACCGGUAACUCCCGACCGGGCUAG 100
SEQ4561_1_100_+    CCACCGGACGACCGGUUCCACCGGUAACUCCCGACCGGGCUAG 100
SEQ5871_2_101_+    CCACCGGACGACCGGUUCCACCGGUAACUCCCGACCGGGCUAG 100
SEQ4492_1_100_+    CCACCGGACGACCGGUUCCACCGGUAACUCCCGACCGGGCUAG 100
SEQ5613_1_102_+    CCACCGGACGACCGGUUCCACCGGUAACUCCCGACCGGGCUAG 102
SEQ5787_1_102_+    CCACCGGACGACCGGUUCCACCGGUAACUCCCGACCGGGCUAG 102
SEQ5538_1_102_+    CCACCGGACGACCGGUUCCACCGGUAACUCCCGACCGGGCUAG 102
                   .........70........80........90........100.
```

Supplementary Figure 5. A dicistronic operon with separate promoters and terminators for each gene. The dicistronic operon encodes the Lsm (HVO_2723) and the Rpl37e (HVO_2722) proteins. Experimental data showed that both genes are transcribed from the first promoter upstream of the *lsm* gene (green arrow upstream of the *lsm* gene)(3). The *rpl37e* gene also has an independent promoter located in the *lsm* gene (green arrow therein)(3). Transcription of the *lsm* gene can terminate at the first terminator downstream of the *lsm* gene (red arrow in *rpl37e* gene). A second terminator downstream of the *rpl37e* gene can terminate the dicistronic *rpl37e/lsm* mRNA or the monocistronic *rpl37e* transcript.

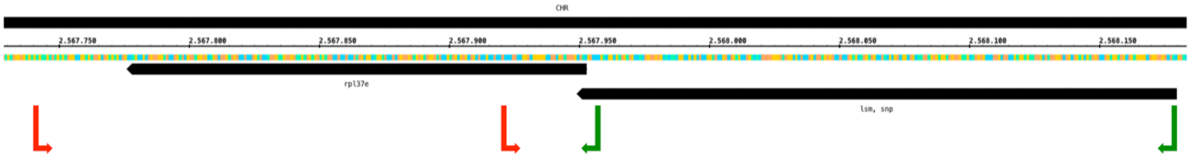

Supplementary Figure 6. Transcripts are not processed at the AGATC motif. *Haloferax* cells were transformed with plasmids containing the AGATC motif, RNA was isolated from cells, separated on denaturing agarose gels and transferred to membranes. Membranes were hybridised with a probe against the sequence downstream of the AGATC motif**. A.** Overview over the type of RNAs generated by termination and **B.** by processing. **A.** If AGATC is used for termination, hybridisation with the downstream probe will detect only the full length transcript that terminates at the vector terminator (424 nucleotides long). **B.** If AGATC is a processing signal, the probe should detect the downstream processing product and the full length transcript (depending on how efficient processing is) (424 nucleotides and approximately 100 nucleotides long). **C.** Northern detection. Lane A1 and A2: RNA isolated from cells with the plasmid containing the A1 and A2 sequence, respectively. The probe detects only the full length transcript (424 nucleotides long), that terminates at the vector terminator. Shorter RNA molecules are not detected (processed product: approximately 100 nucleotides long), showing that the RNA is not processed.

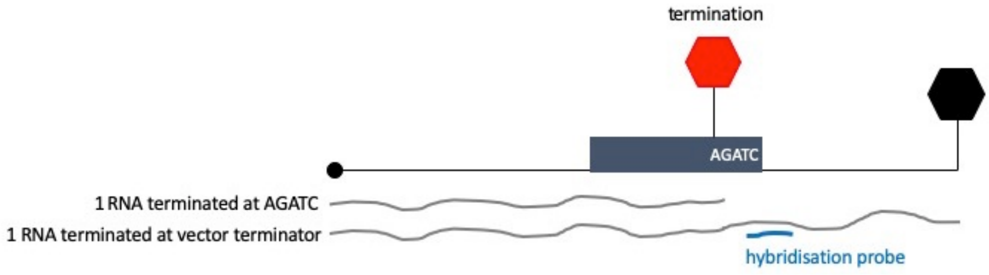

A.

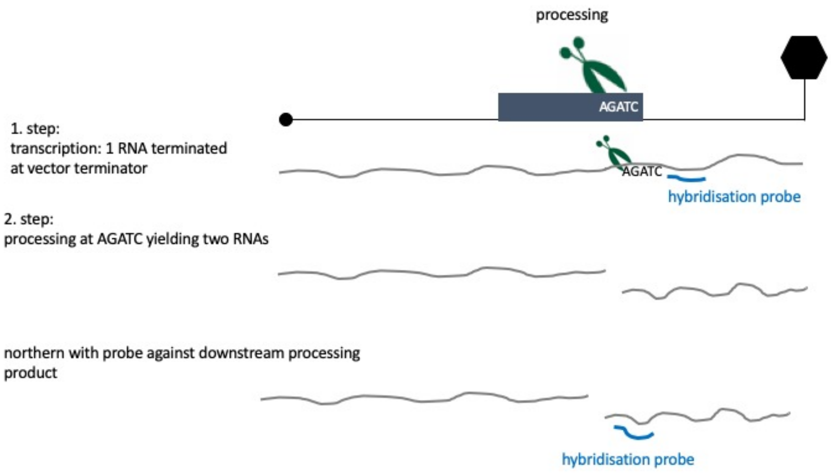

B.

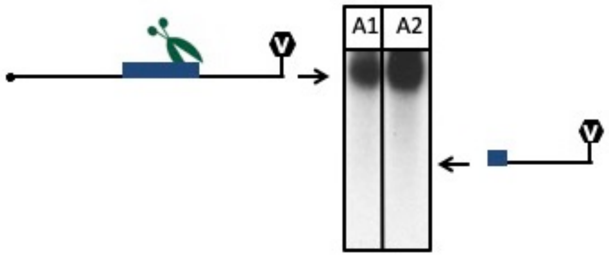

C.

Supplementary Figure 7. Boxplot showing TPM (transcripts per million) ratios for the set of all operons without internal TTS signals (blue) and the set of all operons showing internal TTS signals (red). For each operon the ratio is calculated by dividing the TPM value of the operon's first gene by the TPM value of the operon's last gene. For reasons of visualization, the ratios are shown in logarithmic scale. Outliers on top of the boxes show operons where the TPM value of the first gene is higher than the one of the last gene. Outliers below the box show the opposite, the TPM value of the first gene is lower than the TPM value of the last gene.

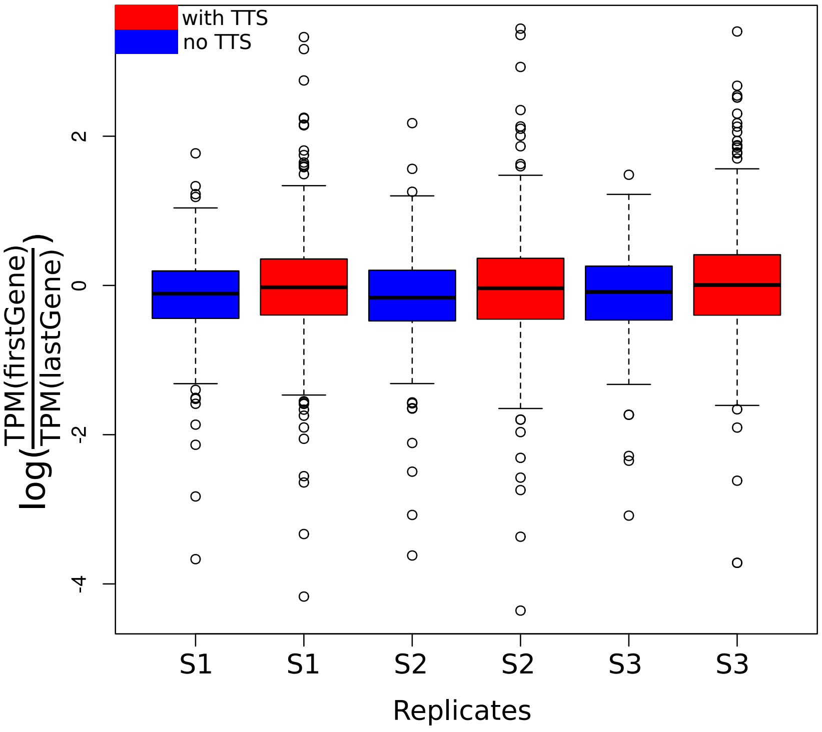

C. Legends to Supplementary Tables 1-4

Supplementary Table 1. Data for all transcription termination sites found with IE-PC. This table contains information corresponding to TTS detected using IE-PC based on the +TEX data (identification of TTS). Columns are as follows:

> **Block General information: #chr:** shows on which chromosome the TTS or RNA 3´ end is located; **TTS:** position of TTS/ RNA 3´ end in genome; **strand**: shows whether the TTS/ RNA 3´ end is on the minus or the plus strand; **usTTS:** distance to closest upstream TTS/ RNA 3´ end, if -1 then there is no other TTS/ RNA 3´ end in between the closest upstream annotated 3´ end and the current TTS/ RNA 3´ end.
>
> **Block Upstream gene: usGene:** distance to closest upstream annotated gene, if -1 there is no upstream gene or information; **usGeneType:** type of upstream gene, e.g. CDS, RNA. If no upstream gene: NA; **usGeneID:** ID of upstream gene as given in the annotation.
>
> **Block TTS/ RNA 3´ end in coding or intergenic region: inGene:** 1 if TTS/ RNA 3´ end is located inside annotated region, 0 if TTS/ RNA 3´ end is located in intergenic regions; **usFive:** distance to closest upstream annotated 5´ end, -1 if no information; **inGeneType:** type of gene the TTS/ RNA 3´ end is located in or NA if intergenic TTS/ RNA 3´ end; **inGeneID:** ID of gene the TTS/ RNA 3´ end is located in or NA if intergenic TTS/ RNA 3´ end.
>
> **Block Sequence motif: seqMotif:** 1 if there is a sequence motif with the TTS/ RNA 3´ end, 0 if not; **motifNum:** number of motif as in paper, 0 if no motif, 1 or 2 for motifs.
>
> **Block 3´ UTR: completeCov:** 1 if complete 3´ UTR is continuously covered with reads from RNAseq, 0 if not; **3´ UTR length:** distance between TTS and closest upstream 3´ end, -1 if no information; **avMeanCov:** mean of average coverage of 3´ UTR by RNAseq reads.
>
> **Block Antisense complete gene: antisenseGene:** 1 if TTS/ RNA 3´ end is located in antisense gene, 0 if not; **antisenseStart:** start position of antisense gene, -1 if no antisense gene; **antisenseEnd:** end position of antisense gene, -1 if no antisense gene; **antisenseID:** ID of antisense gene as given in annotation.

Supplementary Table 2. MEME motif search with data from IE-PC for +TEX reads. A. Motif in coding regions and B. in intergenic regions. Sequence motifs for TTS detected using MEME are listed MEME (see also Figure 8 in main manuscript). For each TTS 15 nt upstream and 5 nt downstream were taken and used as an input sequence for MEME. The table columns are: genome part (CHR, pHV1, pHV3, pHV4), TTS position, start of input region, end of input region, strand, start of motif in input region, p-value.

Supplementary Table 3. RNA 3´ ends discovered using DSM. The table displays all RNA 3´ ends found with DSM. Columns: chromosome: type of chromosome; RNA 3´ end: position of the RNA 3´ end; corresponding average_insert_length: insert length of corresponding reads; strand: lists whether the RNA 3´ end is located on the plus or minus strand.

Supplementary Table 4. 3´ UTRs covered by RNAseq data for RNA 3´ end data. This table contains 10 columns which are also contained in Supplementary Table 1. Here, only 3´ UTRs are listed that are confirmed by continuous coverage of RNAseq reads.

Columns are:

Block General information: #chr: shows on which chromosome the TTS or RNA 3´ end is located; TTS: position of TTS/ RNA 3´ end in genome; strand: shows whether the TTS/ RNA 3´ end is on the minus or the plus strand; usTTS: distance to closest upstream TTS/ RNA 3´ end, if -1 then there is no other TTS/ RNA 3´ end in between the closest upstream annotated 3´ end and the current TTS/ RNA 3´ end.

Block Upstream gene: usGene: distance to closest upstream annotated gene, if -1 there is no upstream gene or information; usGeneType: type of upstream gene, e.g. CDS, RNA. If no upstream gene: NA; usGeneID: ID of upstream gene as given in the annotation.

Block 3´ UTR: 3´UTRcompleteCov: if 3´ UTR is continuously covered with reads from RNAseq: 1, if not: 0; 3´ UTR length: distance between TTS and closest upstream 3´ end, -1 if no information; avMeanCov: mean of average coverage of 3´ UTR by RNAseq reads.

D. Supplementary Tables 5-11

Supplementary Table 5. Primers, synthetic DNA, plasmids and strains used.

a. Strains

strain	genotype	reference
E.coli DH5a	F^-, φ80d*lacZ*ΔM15, Δ(*lacZYA-argF*)U169, *deoR*, *recA*1, *endA*1, *hsdR*17(r_K^-, m_K^+), *phoA*, *supE*44,λ^-, *thi*-1, *gyrA*96, *relA*1	Stratagene
H. volcanii H119	DS70(ΔpHV2), Δ*pyrE*2, Δ*trpA*, Δ*leuB*	Allers *et al*., 2004 (4)
H. volcanii HV55	DS70(ΔpHV2), Δ*pyrE*2, Δ*trpA*, Δ*leuB*, ΔHVO_A0326	this work

b. Plasmids

plasmid		reference
pTA231	ColE1 ori, f1 ori, lacZ, Amp^R, *trpA*, pHV2 ori	Allers et al., 2004 (4)
pTA617	ColE1 ori, f1 ori, lacZ, Amp^R, *pyrE2*, flanking regions HVO_A0326	Large et al., 2007 (5)

c. Synthetic DNA and inserts for termination test

name	sequence
**synthetic DNA**	
psyn overexpression cassette	GCGGCCGCGAGCTCGGATCCGAGAATCGAAACGCTTATAAGTGCCCCCCGGCTA GAGAGCTGCATATGCTCGAGTCTAGATGAGCACCTCTGGACCATCGCATTTTTCG GCGTAAGCTTGGTACCGATATCGAATTC
**inserts for termination tests**	
bgaHa Cterminal fragment	CTCGTGACAGAGTTACTGGACGCCGCCGGCGTCGAGTACACTGAGCGATTCCCG GACGGCGTGCGCGTGATGGAGCGCGACGGCTATACGTGGGCGCTTAACTTCACG AGCGACCCGGTGACGTTGACCGTCCCCGATTCCACCGGGTTCCTGCTCGGTGAG TCCACCGTCGACGCGTTCGATACCGCGGTACTCGACGGATCCATCCGAGGTGTC GGACTCGCGTCCGAGTGAGTCGGCACGGGACCACTAGTTCTAGAGCGGCCGCCA CCGCGGTGGAGCTCCAGCTTTTGTTCCCTTTAGTGAGGGTTAATTGCGCGCTTGG CGCTCGAGATATAA
Control	GGATCCGGCCGAGGCTGGAATCGAATACGTTCGAATGGGGGAGTTCGCGTGGCG ACGAATCGAACCGGAGCGAGGGACGTTCGATTTCGCGTGGTTAGACGAGACCGT CGAACTCATCGGGAAGTCGGATCC
TTS-A1	GGATCCGCACGGCCACCTCGACCACATCGGTGCCATCTCCAAGCTCGCCCACCG CTACGACGCCCCCGTCGTGGCGACGCCCTTCACCATCGAACTGGTGAAACAGCA GATCGAAGGCGAGAACAAGGATCC
TTS-A2	GGATCCCGGCGAGGTCGAAGACGCCTTCGACGCCGGCGTCGTCGAGACGGCCC ACGCCAAAGAGCAGGCCGTCGCCTCCGCCTCCGAAGCCGCGAACCTCGTCCTCA AGATCGACGACATCATCGCGGGATCC
TTS-S12	GGATCCCTGAAGATGTGTCCAACAACCAGCAAACAGACGATGAAATCCACGAGGA CCAGCTCCTTAACTTCCTCGTCAACTCTCTTGACGAGGAAGTTGCTCTCTCACTCG CTGAAAACGCTGAACTGGATCC

TTS-ftsZ2	GGATCCGACGGCGGCCGCGACGAAGTCGAGAAGAACAACGGTCTCGACGTCATC CGGTAACGCCCTGTCCGACCCGCGCGGACGCCGCCGCGCCCGTCGGGTTTCGT GGGTTCTTTTTCGCTGACGTGGATCC

d. Primers

**name**	**sequence**
***bga*HDOprobe**	
bgaHKODO-for	GTCGGCACGGGACCACTC
bgaHKODO-rev	GACGGTGTACACGTGGAG
**bgaHa C-terminal fragment**	
bgaHatermi fw	CTCGTGACAGAGTTACTGGACGCC
bgaHatermi rev	TTATATCTCGAGCGCCAAGCGCGCAATTAAC
**terminator fragments**	
BamHI con fw	TTATATGGATCCGGCCGAGGCTGGAAT
TTS-A1fw	TTATAA GGATCCGCACGGCCACCTCGAC
TTS-A1rev	TTATAA GGATCC CTTGTTCTCGCCTTCG
TTS-A2fw	TTATAAGGATCCCGGCGAGGTCGAAGAC
TTS-A2rev	TTATAAGGATCCCGCGATGATGTCGTC
TermTestS12 fw	TTATATGGATCCCTGAAGATGTGTCCAAC
TermTestS12 rev	TTATATGGATCCAGTTCAGCGTTTTCAGC
TTS-ftsz fw	TTATATGGATCCGACGGCGGCCGCGACGAAGTC
TTS-ftsZ rev	TTATATGGATCCACGTCAGCGAAAAAGAACC
con2rev	ATATAAGGATCCGACTTCCCGATGAGTTC
**probes for northerns**	
TermiVectorrev	GTCGAGTACCGCGGTATCGAAC
BetaHinten1	CGAGGTGTCGGACTCGCGTCC
BetaHinten2	CGAGCGCCAAGCGCGCAATTAACC

Supplementary Table 6. Total reads, mapped and unmapped reads. Three different types of RNAseq data were obtained: -TEX, +TEX and transcriptome data, each with three replicas. The number of reads, uniquely mapped reads and unmapped reads is listed as well as the percentage of mapped reads.

sample	replica	total reads	uniquely mapped	un-mapped	% of mapped reads
-TEX	S1	42,814,712	40,848,267	1,966,445	95
	S2	36,105,224	34,466,376	1,638,848	95
	S3	47,288,670	45,649,822	1,638,848	97
+TEX	S1	52,511,146	46,773,468	5,737,678	89
	S2	32,123,046	29,191,284	2,931,762	91
	S3	37,612,794	34,786,898	2,825,896	92
transcriptome	S1	44,560,765	42,626,326	1,934,439	96
	S2	42,013,751	40,447,008	1,566,743	96
	S3	39,544,091	38,733,269	810,822	98

Supplementary Table 7. Number of RNA 3´ ends determined using DSM. The *Haloferax* genome consists of the main chromosome (2,848 kb) and three chromosomal plasmids pHV1, pHV3 and pHV4 (85, 438 and 636 kb, respectively) (6). RNA 3´ ends are listed for each chromosome separately. In addition, the table shows whether the RNA 3´ ends are located in coding or in intergenic regions.

chromosome	intergenic	coding	total
main	2,046	378	2,424
pHV1	70	22	92
pHV3	204	18	222
pHV4	353	64	417
total	2,673	482	3,155

Supplementary Table 8. Determination of termination efficiencies. Results of quantification of northern blot signals for the different terminator constructs are listed.Total RNA was isolated from strains transformed with the respective reporter gene plasmid. After size separation and transfer, membranes were hybridised with a probe against the β-galactosidase mRNA and signals were detected using phosphorimaging plates. For each lane, signals for termination at the vector terminator and the insert terminator were quantified and their sum set to 100 %; in columns "% vector length" and "% insert length" percentages for each transcript are given per lane. The row indicated by the sample name in bold gives the mean value of the three replicas (samples 1-3) assessed for each terminator motif and the column "SD" gives the standard deviation.

Sample	% vector terminator	% insert terminator	SD
Con -1	99,7	0,3	
Con -2	99,5	0,5	
Con -3	98,8	1,2	
**Control**	**99,3**	**0,7**	**0,5**
FtsZ -1	3,7	96,3	
FtsZ -2	6,1	93,9	
FtsZ -3	5,0	95,1	
**FtsZ**	**4,9**	**95,1**	**1,2**
A1 -1	90,6	9,4	
A1 -2	87,6	12,4	
A1 -3	87,9	12,2	
**A1**	**88,7**	**11,3**	**1,7**
A2 -1	73,0	27,0	
A2 -2	72,2	27,8	
A2 -3	73,9	26,2	
**A2**	**73,0**	**27,0**	**0,8**
S12 -1	41,7	58,3	
S12 -2	36,7	63,3	
S12 -3	42,3	57,7	
**S12**	**40,2**	**59,8**	**3,1**

Supplementary Table 9. Data about *Haloferax* operons. A. Summary of operon information. The number of operons and the number of genes per operon as the maximum, median and minimum for all operons is shown (columns "no of operons", "max no genes", "med no genes", "min no genes"). The last column shows the median length of operons (in bp).

A.

chromosome	no. of operons	max no. genes	med no. genes	min no. genes	median length
main	442	25	2	2	1,742
pHV1	16	4	2	2	854
pHV3	58	11	2	2	2,276
pHV4	116	6	2	2	1,597

B. Detailed operon information. For each operon detailed information is shown. ID: operon number; Start: start of the operon (start of first annotated gene); End: end of the operon (end of last annotated gene); No. genes: number of genes in the operon; TotLen: length of operon from start to end; strand: strand on which the genes are encoded; inTSS: number of TSS located between start and end; inTTS: number of TTS located between start and end; Upstream TSS: number of TSS located upstream of start such that no other annotation on the same strand are between TSS and start; Downstream TTS: Number of TTS downstream of end such that no other annotations on the same strand are between end and the TTS. For the operon detection transcriptome data were used.

ID	Start	End	No. genes	TotLen	strand	inTSS	inTTS	Upstream TSS	Downstream TTS
CHR0	11897	14871	2	2974	-	0	1	0	0
CHR1	19512	22379	2	2867	-	0	0	0	1
CHR10	63622	67652	2	4030	+	0	2	0	0
CHR100	617591	622942	3	5351	+	2	15	1	1
CHR101	627962	629456	4	1494	-	0	5	0	4
CHR102	632375	633620	2	1245	+	0	1	1	1
CHR103	634430	637649	3	3219	-	0	1	0	0
CHR104	638458	639269	3	811	-	0	0	0	0
CHR105	644717	645344	2	627	+	0	5	0	3
CHR106	650251	651967	2	1716	-	1	0	0	1
CHR107	653749	654895	2	1146	-	0	2	0	1
CHR108	656079	657550	2	1471	+	2	1	0	3
CHR109	658118	659073	2	955	-	0	3	1	1
CHR11	76230	80163	4	3933	-	0	1	0	1
CHR110	660288	663036	3	2748	-	1	1	0	1
CHR111	667174	672030	4	4856	+	0	0	0	0
CHR112	672085	676859	4	4774	+	2	0	0	0
CHR113	676918	678230	2	1312	+	0	0	0	0
CHR114	682316	684533	3	2217	+	1	0	0	2
CHR115	687866	688509	2	643	-	0	1	0	1
CHR116	700343	702334	3	1991	-	0	3	1	1
CHR117	702504	705614	2	3110	+	0	16	0	0
CHR118	706709	710387	4	3678	+	1	2	0	2
CHR119	715844	717192	2	1348	+	0	0	0	1

CHR12	83382	85127	2	1745	-	0	1	0	4
CHR120	722661	724015	2	1354	-	1	2	0	1
CHR121	739212	740605	2	1393	+	1	4	1	2
CHR122	746707	749566	3	2859	+	2	3	0	3
CHR123	749624	751158	2	1534	+	0	3	1	2
CHR124	751246	752347	2	1101	+	0	2	1	1
CHR125	752362	753905	2	1543	-	0	1	0	1
CHR126	756983	760286	6	3303	-	1	13	1	3
CHR127	764328	769037	4	4709	+	0	1	0	6
CHR128	769063	770085	2	1022	-	1	0	0	6
CHR129	774651	778211	3	3560	+	0	20	0	4
CHR13	85507	87266	2	1759	-	0	0	0	0
CHR130	778355	779303	2	948	+	0	5	1	6
CHR131	780439	781164	2	725	+	3	1	1	0
CHR132	791288	791784	2	496	+	0	0	0	0
CHR133	802107	802476	2	369	+	0	0	0	0
CHR134	802517	805137	2	2620	-	1	8	1	4
CHR135	806141	809728	3	3587	-	0	2	1	7
CHR136	818606	822247	4	3641	+	1	0	0	0
CHR137	827150	828747	2	1597	+	2	4	1	1
CHR138	835405	836572	2	1167	+	0	0	0	0
CHR139	847700	850499	2	2799	+	0	1	1	0
CHR14	88714	89226	3	512	+	0	0	0	1
CHR140	851051	851878	2	827	-	0	2	1	0
CHR141	851945	855442	3	3497	-	0	4	0	1
CHR142	855608	858335	4	2727	+	0	5	0	3
CHR143	861842	864859	3	3017	-	1	1	1	1
CHR144	864943	866869	3	1926	-	0	0	0	0
CHR145	868340	870492	4	2152	+	1	2	1	3
CHR146	883706	885261	2	1555	-	1	0	0	2
CHR147	887289	889052	2	1763	+	1	0	0	2
CHR148	889252	893543	5	4291	+	1	17	1	1
CHR149	893649	899663	6	6014	+	6	7	1	0
CHR15	100055	101548	2	1493	+	0	5	0	2
CHR150	902466	902894	2	428	-	1	0	0	0
CHR151	908202	911458	2	3256	-	0	2	1	4
CHR152	914365	916070	2	1705	-	0	0	0	1
CHR153	920072	920722	2	650	-	0	1	1	0
CHR154	920789	922882	2	2093	-	1	3	1	2
CHR155	923927	924837	2	910	-	0	2	1	2
CHR156	933893	935421	2	1528	-	2	0	0	3
CHR157	941377	945196	2	3819	-	0	0	0	0
CHR158	949985	951300	2	1315	+	0	1	0	1
CHR159	956966	958740	2	1774	+	0	3	1	1
CHR16	103089	104658	2	1569	+	0	0	0	2
CHR160	960564	962114	2	1550	+	0	2	1	3
CHR161	962244	964585	2	2341	+	0	2	0	1
CHR162	971509	977696	7	6187	-	2	1	0	1
CHR163	983588	984540	2	952	-	1	0	0	0
CHR164	986942	988644	2	1702	-	1	0	0	4
CHR165	996181	997312	2	1131	+	1	0	0	2
CHR166	997547	1000106	4	2559	-	2	8	1	2
CHR167	1000850	1006589	6	5739	-	1	3	1	0

CHR168	1012676	1013178	2	502	-	0	1	0	2
CHR169	1014484	1017005	2	2521	+	0	0	0	1
CHR17	108419	109462	2	1043	-	0	1	0	6
CHR170	1019071	1019618	2	547	-	0	1	1	3
CHR171	1021329	1022863	2	1534	+	1	1	0	2
CHR172	1028819	1030869	2	2050	+	0	1	0	0
CHR173	1045827	1048314	4	2487	-	0	7	1	2
CHR174	1049190	1050838	3	1648	+	1	10	1	3
CHR175	1052529	1053748	2	1219	-	0	0	0	3
CHR176	1053802	1061259	5	7457	-	1	2	1	0
CHR177	1063656	1064809	2	1153	-	0	0	0	2
CHR178	1070635	1072603	3	1968	+	1	2	1	2
CHR179	1084301	1088844	6	4543	+	1	0	0	0
CHR18	109554	110752	2	1198	+	0	1	0	1
CHR180	1088887	1090161	2	1274	-	2	6	1	1
CHR181	1095684	1096415	2	731	-	0	0	0	0
CHR182	1096507	1098590	3	2083	-	0	0	0	0
CHR183	1100086	1101400	2	1314	+	1	0	0	1
CHR184	1102755	1110353	7	7598	+	3	1	0	0
CHR185	1110849	1115934	4	5085	-	1	0	0	2
CHR186	1121664	1122844	2	1180	-	0	0	0	0
CHR187	1132536	1134265	2	1729	-	0	2	0	2
CHR188	1135740	1137022	2	1282	-	0	1	0	2
CHR189	1139167	1139841	2	674	+	0	2	0	4
CHR19	110903	112006	3	1103	+	0	3	0	1
CHR190	1141388	1141840	2	452	-	0	0	0	0
CHR191	1143500	1151204	6	7704	-	2	3	1	0
CHR192	1152937	1154183	2	1246	-	1	0	0	0
CHR193	1154320	1155953	2	1633	+	1	1	1	2
CHR194	1157407	1159534	2	2127	+	0	2	0	1
CHR195	1163765	1164931	2	1166	+	0	4	0	1
CHR196	1168624	1171870	4	3246	-	1	6	1	1
CHR197	1176818	1179008	2	2190	+	0	1	0	2
CHR198	1179103	1181293	3	2190	+	0	1	1	1
CHR199	1188454	1191291	2	2837	-	0	12	0	3
CHR2	28190	30712	2	2522	+	0	4	0	3
CHR20	112088	114137	3	2049	+	0	8	0	7
CHR200	1204459	1205636	2	1177	-	1	0	0	1
CHR201	1205784	1206448	2	664	+	0	0	0	3
CHR202	1214772	1215322	2	550	+	1	0	0	0
CHR203	1215368	1219306	3	3938	-	0	2	0	1
CHR204	1225438	1227398	2	1960	-	0	1	0	1
CHR205	1239765	1240954	2	1189	-	2	0	0	0
CHR206	1241450	1243590	3	2140	-	1	0	0	0
CHR207	1243858	1244687	2	829	+	0	0	0	2
CHR208	1246610	1249608	3	2998	-	0	1	0	3
CHR209	1255092	1255854	2	762	+	0	1	0	1
CHR21	114180	115805	2	1625	-	0	0	0	2
CHR210	1259125	1260236	2	1111	+	0	2	0	3
CHR211	1263156	1264767	2	1611	-	1	0	0	2
CHR212	1265949	1267759	2	1810	-	0	0	0	2
CHR213	1272027	1275951	3	3924	-	2	5	1	2
CHR214	1291354	1291661	2	307	+	0	0	0	1

CHR215	1294150	1294863	2	713	+	0	0	0	1
CHR216	1295035	1296386	2	1351	-	0	4	1	2
CHR217	1300408	1304013	5	3605	-	2	3	1	0
CHR218	1304083	1304858	2	775	-	0	1	0	3
CHR219	1311468	1315379	3	3911	-	0	1	1	0
CHR22	117403	118545	2	1142	+	0	1	0	0
CHR220	1326226	1327604	2	1378	+	1	3	1	1
CHR221	1332326	1334325	2	1999	-	0	0	0	0
CHR222	1334502	1336671	2	2169	-	0	0	0	1
CHR223	1337856	1338846	2	990	+	0	0	0	2
CHR224	1346000	1347021	2	1021	-	0	0	0	0
CHR225	1351155	1351968	2	813	+	1	0	0	2
CHR226	1352530	1353307	2	777	-	0	0	0	0
CHR227	1356016	1357814	2	1798	+	0	0	0	0
CHR228	1364228	1367135	4	2907	-	1	3	1	4
CHR229	1368413	1370094	2	1681	-	1	0	0	4
CHR23	119674	120662	2	988	-	0	1	0	0
CHR230	1370298	1377018	7	6720	-	1	7	0	1
CHR231	1379388	1380610	2	1222	+	1	0	0	2
CHR232	1383031	1385466	3	2435	+	1	2	1	1
CHR233	1386375	1388401	2	2026	-	3	3	1	0
CHR234	1405185	1409507	3	4322	+	1	3	1	5
CHR235	1409871	1412471	2	2600	+	1	6	1	1
CHR236	1412928	1414009	2	1081	-	0	1	1	2
CHR237	1423128	1425433	2	2305	+	1	0	0	2
CHR238	1437643	1441139	2	3496	-	0	8	1	3
CHR239	1441393	1445902	2	4509	+	0	5	1	4
CHR24	121710	122181	2	471	+	0	0	0	2
CHR240	1446425	1447794	2	1369	-	1	3	1	8
CHR241	1451074	1453520	3	2446	-	2	0	0	1
CHR242	1465704	1467178	2	1474	+	1	0	0	3
CHR243	1470929	1474137	4	3208	-	2	2	1	1
CHR244	1484423	1485328	2	905	+	0	0	0	0
CHR245	1486496	1487981	2	1485	+	0	0	0	0
CHR246	1499837	1501104	2	1267	+	1	2	0	0
CHR247	1502585	1504810	5	2225	+	0	0	0	0
CHR248	1506162	1510012	4	3850	-	6	1	1	0
CHR249	1523528	1525235	2	1707	-	0	1	1	0
CHR25	131762	133107	2	1345	+	0	0	0	0
CHR250	1526812	1527509	2	697	-	1	3	1	1
CHR251	1529404	1530902	2	1498	-	0	0	0	1
CHR252	1531145	1531977	2	832	+	0	0	0	1
CHR253	1532792	1533927	2	1135	+	0	0	0	3
CHR254	1548294	1550991	2	2697	-	1	0	0	3
CHR255	1562255	1563230	2	975	-	0	0	0	2
CHR256	1567095	1573376	4	6281	+	1	12	1	1
CHR257	1587408	1588657	2	1249	-	0	1	0	1
CHR258	1603637	1604640	2	1003	+	1	7	1	10
CHR259	1607341	1610092	2	2751	+	0	8	1	5
CHR26	135168	136798	2	1630	+	0	0	0	3
CHR260	1610100	1611874	3	1774	-	2	5	1	4
CHR261	1613635	1616816	2	3181	-	2	4	1	3
CHR262	1626783	1629765	3	2982	+	0	3	1	1

CHR263	1633851	1642469	11	8618	+	1	0	0	5
CHR264	1647441	1648317	2	876	+	1	0	0	1
CHR265	1651680	1651978	2	298	-	0	1	0	3
CHR266	1657952	1660058	5	2106	-	0	0	0	0
CHR267	1662369	1663783	2	1414	-	0	1	0	0
CHR268	1668011	1669796	2	1785	-	0	0	0	0
CHR269	1682896	1683815	2	919	+	0	2	1	2
CHR27	137039	142417	7	5378	+	0	1	0	1
CHR270	1686807	1687670	2	863	-	0	2	1	2
CHR271	1694289	1696397	2	2108	-	3	0	0	0
CHR272	1697021	1698178	3	1157	-	0	2	0	0
CHR273	1715144	1716100	2	956	-	1	5	1	1
CHR274	1721045	1722199	2	1154	-	1	1	1	0
CHR275	1722293	1724675	2	2382	+	0	2	1	2
CHR276	1730131	1731374	2	1243	-	0	1	1	3
CHR277	1740018	1742632	3	2614	-	1	0	0	1
CHR278	1742773	1743340	2	567	+	0	0	0	0
CHR279	1743400	1743746	2	346	-	1	0	0	0
CHR28	148866	149722	2	856	-	0	2	0	3
CHR280	1745405	1747402	2	1997	-	0	8	0	2
CHR281	1747471	1750427	5	2956	-	2	9	1	3
CHR282	1752587	1753620	2	1033	-	0	0	0	1
CHR283	1758755	1759386	2	631	+	0	0	0	0
CHR284	1759649	1761608	3	1959	+	0	0	0	1
CHR285	1768353	1770297	2	1944	+	1	1	0	4
CHR286	1780813	1781150	2	337	-	0	0	0	0
CHR287	1785835	1790288	2	4453	-	0	0	0	1
CHR288	1790495	1794722	3	4227	+	0	1	1	2
CHR289	1797762	1799752	4	1990	-	0	0	0	0
CHR29	164618	165506	2	888	-	0	1	0	1
CHR290	1800526	1803762	2	3236	-	0	2	0	0
CHR291	1813650	1814372	2	722	+	0	3	1	8
CHR292	1817248	1819327	3	2079	-	0	0	0	0
CHR293	1821227	1823661	2	2434	+	3	5	1	1
CHR294	1828262	1828856	2	594	-	0	0	0	0
CHR295	1829072	1831403	2	2331	-	0	0	0	0
CHR296	1832000	1833735	2	1735	+	1	0	0	2
CHR297	1833881	1835816	2	1935	+	3	2	1	1
CHR298	1844851	1846637	2	1786	-	0	1	0	0
CHR299	1872299	1874475	3	2176	+	1	2	1	0
CHR3	30936	32206	2	1270	+	0	1	0	0
CHR30	180588	181207	2	619	-	0	0	0	1
CHR300	1876480	1880227	3	3747	+	1	1	0	2
CHR301	1893837	1895808	2	1971	+	3	6	1	5
CHR302	1908182	1909585	2	1403	+	0	2	1	2
CHR303	1917415	1921572	2	4157	-	5	11	1	3
CHR304	1932315	1932884	2	569	+	0	0	1	0
CHR305	1933890	1937793	2	3903	-	5	5	1	7
CHR306	1941436	1944444	3	3008	+	3	4	1	3
CHR307	1964595	1965069	2	474	-	0	2	0	0
CHR308	1967896	1969806	3	1910	-	1	0	0	0
CHR309	1974142	1976387	2	2245	-	1	1	0	2
CHR31	196563	198541	3	1978	-	0	0	0	0

CHR310	1980207	1982068	2	1861	+	0	0	0	3
CHR311	1982189	1984137	2	1948	+	0	3	0	2
CHR312	1985891	1990238	5	4347	-	1	2	0	1
CHR313	1993640	1996334	3	2694	+	1	0	0	1
CHR314	2033868	2037127	3	3259	+	0	0	0	0
CHR315	2037202	2038389	2	1187	+	0	0	0	0
CHR316	2039493	2042470	3	2977	-	0	0	0	0
CHR317	2046170	2048817	2	2647	+	0	0	0	0
CHR318	2049729	2051804	5	2075	+	0	1	0	1
CHR319	2057607	2058534	2	927	-	0	0	0	1
CHR32	202030	203306	2	1276	+	0	0	0	0
CHR320	2062859	2065668	4	2809	+	0	0	0	0
CHR321	2066232	2068149	2	1917	+	0	0	0	0
CHR322	2070415	2072992	3	2577	-	0	11	1	1
CHR323	2077064	2077986	2	922	+	1	1	1	2
CHR324	2081654	2081925	2	271	-	0	0	0	0
CHR325	2082166	2082735	2	569	-	1	0	0	1
CHR326	2094572	2096085	2	1513	+	0	2	0	2
CHR327	2096172	2097303	2	1131	+	0	1	0	0
CHR328	2097818	2098575	2	757	-	2	0	0	1
CHR329	2103547	2104810	3	1263	-	1	3	1	0
CHR33	203358	204871	2	1513	+	0	0	0	1
CHR330	2117186	2118960	3	1774	+	1	0	0	1
CHR331	2119332	2119757	2	425	+	0	0	0	4
CHR332	2122933	2125901	3	2968	+	2	3	0	0
CHR333	2126758	2131603	3	4845	+	6	2	0	0
CHR334	2131833	2133417	3	1584	+	4	0	0	1
CHR335	2140967	2148052	8	7085	-	4	2	1	0
CHR336	2168213	2169418	2	1205	-	0	2	1	0
CHR337	2169999	2172052	2	2053	-	1	4	0	0
CHR338	2172194	2175315	2	3121	+	0	0	0	1
CHR339	2176949	2177616	2	667	-	0	2	0	1
CHR34	209051	209917	2	866	-	0	3	0	3
CHR340	2178257	2181349	3	3092	-	0	5	1	2
CHR341	2185692	2187457	2	1765	+	1	0	0	0
CHR342	2190129	2191843	2	1714	+	0	0	0	0
CHR343	2192908	2194300	2	1392	+	2	1	0	1
CHR344	2207083	2209223	2	2140	+	1	1	0	1
CHR345	2240557	2244046	3	3489	+	4	1	0	1
CHR346	2252319	2260621	4	8302	+	1	0	0	0
CHR347	2264658	2265427	2	769	-	0	1	0	2
CHR348	2265616	2268583	3	2967	+	0	3	0	0
CHR349	2268958	2273198	4	4240	-	0	2	0	2
CHR35	213507	215811	2	2304	+	0	1	0	2
CHR350	2273429	2275978	4	2549	-	1	0	0	0
CHR351	2276649	2280555	2	3906	-	0	0	0	0
CHR352	2297996	2299551	2	1555	-	1	2	1	2
CHR353	2308617	2308964	2	347	+	0	0	0	0
CHR354	2310076	2314654	7	4578	+	0	1	0	2
CHR355	2318839	2319735	2	896	-	1	4	0	1
CHR356	2324131	2327975	4	3844	-	0	0	0	0
CHR357	2329410	2332152	2	2742	-	0	0	0	1
CHR358	2332664	2334675	2	2011	-	0	4	1	2

CHR359	2341670	2343486	2	1816	+	2	0	0	0
CHR36	223693	225164	2	1471	+	0	0	0	0
CHR360	2344719	2348261	5	3542	-	1	0	0	2
CHR361	2357212	2357734	2	522	+	0	0	0	0
CHR362	2359296	2360746	2	1450	-	1	3	1	1
CHR363	2364709	2367422	2	2713	+	0	0	0	1
CHR364	2372097	2373072	2	975	-	0	2	1	1
CHR365	2376033	2378199	2	2166	-	1	2	1	3
CHR366	2392420	2395730	3	3310	-	1	0	0	0
CHR367	2398798	2400158	2	1360	-	1	2	1	3
CHR368	2403719	2405013	2	1294	-	0	1	0	1
CHR369	2406014	2420295	25	14281	-	4	66	1	2
CHR37	226809	227578	2	769	+	0	0	0	0
CHR370	2422082	2423464	2	1382	-	0	1	1	1
CHR371	2424275	2425695	2	1420	+	0	0	0	0
CHR372	2430345	2433905	3	3560	-	0	0	0	1
CHR373	2444152	2446545	3	2393	+	0	0	0	1
CHR374	2455085	2457615	2	2530	+	0	0	0	2
CHR375	2457666	2458664	2	998	-	1	0	0	0
CHR376	2462016	2464178	2	2162	+	0	1	0	0
CHR377	2465565	2468568	3	3003	+	1	0	0	1
CHR378	2474617	2477500	3	2883	+	1	4	1	1
CHR379	2480448	2481917	2	1469	+	0	1	0	1
CHR38	229170	231071	2	1901	+	0	0	0	0
CHR380	2487257	2489320	2	2063	-	0	1	0	0
CHR381	2491040	2492390	2	1350	-	1	1	1	2
CHR382	2501833	2505040	4	3207	-	0	3	1	1
CHR383	2514799	2517176	3	2377	-	0	0	0	1
CHR384	2526511	2527164	2	653	+	1	3	1	2
CHR385	2527654	2529053	2	1399	+	1	2	0	0
CHR386	2533589	2534329	2	740	+	0	2	0	1
CHR387	2537992	2541168	3	3176	-	1	6	0	1
CHR388	2544015	2546659	3	2644	+	1	6	1	2
CHR389	2546945	2549461	2	2516	-	1	7	1	3
CHR39	231538	232742	2	1204	-	0	0	0	0
CHR390	2553361	2555451	2	2090	-	0	1	1	2
CHR391	2556718	2559328	2	2610	+	1	1	1	0
CHR392	2562785	2564538	2	1753	-	0	9	0	4
CHR393	2567777	2568180	2	403	-	0	1	0	3
CHR394	2568561	2570959	2	2398	+	0	7	0	1
CHR395	2574770	2579517	4	4747	+	0	0	0	0
CHR396	2583344	2584605	4	1261	+	0	4	0	1
CHR397	2584975	2587055	2	2080	-	0	2	0	1
CHR398	2587115	2589768	3	2653	-	0	3	1	0
CHR399	2590458	2591108	2	650	-	0	3	1	3
CHR4	41076	44334	3	3258	-	0	0	0	1
CHR40	233415	234040	2	625	+	0	0	0	0
CHR400	2592836	2593284	2	448	-	0	1	0	2
CHR401	2594860	2596904	3	2044	-	1	8	1	2
CHR402	2599558	2601404	2	1846	+	0	3	0	0
CHR403	2602293	2604394	2	2101	+	1	0	0	0
CHR404	2610067	2610644	2	577	+	0	1	0	1
CHR405	2614266	2617805	7	3539	-	1	23	1	1

CHR406	2618061	2620263	4	2202	-	0	13	0	1
CHR407	2623372	2624738	2	1366	+	0	3	1	2
CHR408	2631348	2635402	4	4054	-	1	1	0	0
CHR409	2639676	2641323	3	1647	-	2	3	1	6
CHR41	237481	237988	2	507	+	0	0	0	2
CHR410	2655111	2656568	3	1457	+	1	3	1	0
CHR411	2656634	2662204	3	5570	+	3	2	1	1
CHR412	2667268	2668163	2	895	-	0	1	0	1
CHR413	2682183	2690404	5	8221	-	1	4	1	0
CHR414	2690938	2691792	2	854	+	1	0	0	1
CHR415	2701945	2702813	2	868	+	0	0	0	1
CHR416	2710411	2712036	2	1625	-	0	2	0	0
CHR417	2712095	2713114	2	1019	+	1	0	0	1
CHR418	2716454	2718326	2	1872	-	2	1	0	2
CHR419	2730421	2731619	2	1198	-	0	0	0	3
CHR42	238338	243640	7	5302	+	0	0	0	0
CHR420	2732769	2734452	2	1683	-	0	1	0	0
CHR421	2740705	2742187	2	1482	-	0	3	1	1
CHR422	2742803	2744214	2	1411	-	0	0	0	4
CHR423	2753026	2754801	3	1775	+	0	0	0	0
CHR424	2756256	2757896	2	1640	+	0	5	1	1
CHR425	2757995	2759152	3	1157	+	0	0	0	0
CHR426	2764451	2765257	2	806	+	0	1	0	1
CHR427	2779920	2783131	2	3211	-	1	10	1	2
CHR428	2785575	2788180	2	2605	-	1	2	0	1
CHR429	2794100	2799182	4	5082	+	0	9	0	4
CHR43	245331	247094	2	1763	+	0	2	0	0
CHR430	2799214	2799839	2	625	-	0	0	0	2
CHR431	2807898	2808928	2	1030	+	0	0	0	0
CHR432	2810921	2812184	2	1263	-	0	2	0	0
CHR433	2812286	2813841	2	1555	+	1	0	0	2
CHR434	2822983	2824590	2	1607	-	2	2	1	0
CHR435	2828360	2830923	2	2563	-	0	0	0	0
CHR436	2833318	2835218	2	1900	-	0	1	1	1
CHR437	311653	311827	2	174	-	0	1	0	0
CHR438	2420585	2420738	2	153	+	0	0	0	0
CHR439	2218063	2218293	2	230	+	0	3	0	0
CHR44	251621	251961	2	340	-	0	0	0	0
CHR440	2264510	2264625	2	115	+	0	1	0	0
CHR441	2568379	2568524	2	145	+	0	1	0	0
CHR45	260600	262126	2	1526	-	0	4	0	2
CHR46	272558	274305	2	1747	-	0	5	0	4
CHR47	275700	277613	2	1913	+	0	2	0	2
CHR48	278458	280978	2	2520	+	0	9	0	2
CHR49	281153	286554	6	5401	+	0	22	0	2
CHR5	44389	46485	3	2096	-	0	3	0	1
CHR50	291490	293391	2	1901	+	0	4	0	2
CHR51	296025	297424	2	1399	+	0	4	0	4
CHR52	304760	306147	2	1387	+	0	0	0	2
CHR53	309492	311250	3	1758	+	0	0	0	1
CHR54	312095	320326	6	8231	+	0	25	0	2
CHR55	321385	322430	2	1045	+	0	6	0	9
CHR56	325966	327485	2	1519	+	0	2	0	8

CHR57	327676	329252	2	1576	+	0	10	0	3
CHR58	331222	334589	9	3367	-	0	0	0	0
CHR59	334719	336630	5	1911	+	0	0	0	0
CHR6	46806	49491	2	2685	-	0	4	0	2
CHR60	338106	339210	2	1104	-	0	1	0	4
CHR61	340199	341785	4	1586	+	0	0	0	2
CHR62	361646	363423	2	1777	-	0	2	0	1
CHR63	365370	365955	2	585	+	0	0	0	1
CHR64	365957	367695	2	1738	-	0	0	0	0
CHR65	368086	371091	2	3005	-	0	4	0	1
CHR66	380247	382378	2	2131	+	0	3	0	2
CHR67	384018	385526	2	1508	-	0	0	0	1
CHR68	386169	387507	2	1338	+	0	0	0	0
CHR69	392044	393052	2	1008	-	0	1	0	1
CHR7	49976	51675	2	1699	-	0	0	0	0
CHR70	393182	394581	3	1399	+	0	2	0	0
CHR71	394719	396477	2	1758	-	0	1	0	2
CHR72	409132	411793	3	2661	-	0	4	0	2
CHR73	422870	424564	2	1694	-	0	2	0	3
CHR74	447374	448179	2	805	-	0	1	0	2
CHR75	458359	459087	2	728	+	0	4	0	1
CHR76	459084	459949	2	865	-	0	1	0	0
CHR77	461838	465227	3	3389	+	0	6	0	4
CHR78	468630	469891	2	1261	-	0	0	0	0
CHR79	481150	486128	2	4978	-	0	0	0	0
CHR8	51719	54959	3	3240	-	0	4	0	1
CHR80	486347	490083	2	3736	+	0	0	0	0
CHR81	492257	493255	2	998	+	0	5	0	1
CHR82	496045	499357	3	3312	-	0	0	0	3
CHR83	502893	506152	2	3259	+	0	1	0	1
CHR84	516141	517531	2	1390	-	0	9	0	7
CHR85	519896	525578	7	5682	+	0	0	0	1
CHR86	527070	528424	2	1354	+	0	1	0	0
CHR87	531046	532526	2	1480	+	0	0	0	1
CHR88	541192	543194	2	2002	+	0	0	0	1
CHR89	545379	552629	7	7250	-	0	0	0	0
CHR9	55732	61083	5	5351	-	0	15	0	2
CHR90	560896	564999	4	4103	+	0	9	0	1
CHR91	565023	569050	2	4027	-	0	0	0	0
CHR92	571195	574485	4	3290	+	0	1	0	0
CHR93	583221	583868	2	647	-	1	5	1	2
CHR94	583912	584621	3	709	-	0	4	1	5
CHR95	587495	589224	2	1729	-	0	1	0	1
CHR96	590190	592441	3	2251	+	0	1	0	1
CHR97	595636	598727	3	3091	-	1	1	0	0
CHR98	605951	606410	2	459	+	0	0	0	0
CHR99	614347	615697	2	1350	+	1	0	0	0
pHV10	5326	5456	3	130	-	0	2	0	0
pHV11	5497	6006	2	509	-	0	2	1	2
pHV110	60310	60872	3	562	+	0	0	0	0
pHV111	62797	63476	3	679	+	0	1	0	0
pHV112	70334	72192	2	1858	-	2	2	1	8
pHV113	79142	79477	2	335	-	0	1	0	1

pHV114	79485	80892	2	1407	-	1	1	0	0
pHV115	83320	84525	4	1205	-	2	1	1	0
pHV12	11678	12409	2	731	-	0	3	0	5
pHV13	12637	13925	2	1288	+	1	0	0	1
pHV14	14579	14834	3	255	+	0	1	0	12
pHV15	18908	20628	3	1720	+	0	6	1	2
pHV16	42448	45461	2	3013	+	3	6	1	1
pHV17	50866	53205	3	2339	-	1	9	0	9
pHV18	54474	54645	3	171	-	0	0	0	0
pHV19	59217	60195	2	978	-	0	1	0	1
pHV30	8220	9729	2	1509	-	2	2	1	0
pHV31	11360	13309	2	1949	+	2	1	0	0
pHV310	73155	74520	3	1365	+	0	6	0	5
pHV311	76732	78275	2	1543	-	1	0	0	1
pHV312	82704	93334	9	10630	-	6	22	1	2
pHV313	100521	102649	2	2128	-	0	2	1	1
pHV314	102695	104724	2	2029	-	0	0	0	2
pHV315	120598	122489	2	1891	+	0	0	0	1
pHV316	125057	127293	2	2236	+	1	5	1	3
pHV317	138313	140242	2	1929	+	0	0	0	0
pHV318	147763	150161	2	2398	-	0	1	0	3
pHV319	152981	155490	2	2509	-	0	0	0	0
pHV32	16218	16822	2	604	-	1	4	1	5
pHV320	155618	157784	2	2166	+	0	0	0	0
pHV321	171961	174764	2	2803	-	3	12	1	1
pHV322	186336	193232	6	6896	+	3	0	0	0
pHV323	193290	197886	5	4596	+	0	0	0	0
pHV324	198487	199328	2	841	-	1	0	0	0
pHV325	204905	208585	3	3680	-	1	0	0	0
pHV326	208868	210395	2	1527	-	0	0	0	0
pHV327	215327	217164	2	1837	+	0	2	0	4
pHV328	217538	221257	4	3719	+	5	2	1	0
pHV329	223140	223824	2	684	-	1	4	0	3
pHV33	16978	18260	2	1282	-	2	0	0	2
pHV330	226697	229012	2	2315	-	2	1	1	0
pHV331	244643	246328	2	1685	-	0	0	0	0
pHV332	246774	249367	2	2593	+	0	2	1	0
pHV333	258281	261871	4	3590	+	1	2	1	0
pHV334	269467	273369	4	3902	+	1	0	0	6
pHV335	279990	284839	2	4849	-	1	1	1	0
pHV336	287729	289350	2	1621	-	0	14	1	3
pHV337	296165	297144	2	979	-	1	0	0	0
pHV338	304696	306480	2	1784	+	1	0	0	0
pHV339	310915	313517	2	2602	-	0	0	0	0
pHV34	22808	26834	3	4026	+	2	0	0	0
pHV340	313647	317768	2	4121	+	2	0	0	2
pHV341	317826	320338	2	2512	-	2	2	1	2
pHV342	323997	324436	2	439	-	0	0	0	0
pHV343	327393	328153	2	760	+	1	0	0	2
pHV344	331999	333530	2	1531	+	1	0	0	0
pHV345	340588	344988	4	4400	+	6	1	1	0
pHV346	346327	347605	2	1278	-	0	1	0	1
pHV347	351603	353365	2	1762	-	0	3	1	0

pHV348	354247	357996	3	3749	+	0	1	0	0
pHV349	358112	359831	7	1719	+	0	0	0	0
pHV35	29918	31956	2	2038	+	1	0	0	0
pHV350	360154	363931	3	3777	-	2	1	1	3
pHV351	375818	378460	2	2642	-	1	0	0	0
pHV352	384446	386619	2	2173	+	0	1	1	0
pHV353	391344	392839	2	1495	-	0	0	0	0
pHV354	410664	414009	3	3345	-	1	3	1	1
pHV355	418592	424082	5	5490	+	3	4	1	1
pHV356	424234	427174	4	2940	+	0	1	0	0
pHV357	433504	436474	3	2970	-	1	0	0	0
pHV36	37352	41546	4	4194	+	2	0	0	2
pHV37	46367	54941	6	8574	-	2	0	0	3
pHV38	56690	62022	3	5332	-	2	10	1	3
pHV39	64223	72985	11	8762	-	5	19	1	9
pHV40	6551	8015	2	1464	+	0	7	0	2
pHV41	8150	9687	2	1537	+	0	4	0	2
pHV410	61404	62656	2	1252	-	0	0	0	3
pHV4100	562540	568159	4	5619	-	0	0	0	1
pHV4101	570870	574873	4	4003	-	0	0	0	1
pHV4102	575069	577125	2	2056	-	1	0	0	2
pHV4103	577192	578567	2	1375	-	0	0	0	0
pHV4104	581819	586675	5	4856	+	1	0	0	0
pHV4105	597454	600440	2	2986	-	0	0	0	0
pHV4106	607809	609364	2	1555	-	1	0	0	0
pHV4107	609848	610833	2	985	-	0	0	0	0
pHV4108	610830	612610	2	1780	-	1	0	0	0
pHV4109	617447	619418	2	1971	-	0	0	0	0
pHV411	62930	64375	2	1445	-	1	1	0	0
pHV4110	619529	620598	2	1069	+	1	1	1	1
pHV4111	624026	625689	2	1663	-	0	3	0	5
pHV4112	631456	633927	2	2471	+	1	1	0	1
pHV4113	129485	129841	2	356	+	0	2	1	0
pHV4114	207662	207873	2	211	+	0	3	0	0
pHV4115	250939	251268	2	329	-	1	4	0	0
pHV412	66855	71505	2	4650	+	4	2	1	10
pHV413	71552	75701	2	4149	-	1	7	0	7
pHV414	83529	85046	2	1517	-	0	1	0	1
pHV415	88508	91754	2	3246	-	1	6	1	4
pHV416	105533	106533	2	1000	-	0	2	0	1
pHV417	109523	110275	2	752	-	0	0	0	0
pHV418	113348	114104	2	756	-	0	1	0	2
pHV419	127679	128602	2	923	+	2	0	0	3
pHV42	10786	12518	2	1732	-	1	3	1	4
pHV420	129784	130371	3	587	-	0	2	0	1
pHV421	130525	130688	2	163	-	0	1	0	0
pHV422	133406	134105	2	699	+	1	0	0	1
pHV423	141079	144250	3	3171	-	7	1	1	2
pHV424	151328	153903	3	2575	-	1	2	1	0
pHV425	163583	165636	2	2053	+	1	0	0	3
pHV426	165667	168516	3	2849	-	2	0	0	2
pHV427	168878	169276	2	398	+	1	0	0	0
pHV428	179508	181339	2	1831	-	0	0	0	0

pHV429	182280	184846	2	2566	+	0	7	1	0
pHV43	30955	31815	2	860	+	0	0	0	3
pHV430	186868	188408	2	1540	+	0	1	0	0
pHV431	189115	189824	2	709	-	0	1	0	2
pHV432	191621	193056	2	1435	+	0	0	0	0
pHV433	207954	215614	5	7660	+	5	17	1	0
pHV434	215804	217616	3	1812	+	1	0	0	2
pHV435	222046	228387	6	6341	+	0	0	0	0
pHV436	229855	231125	2	1270	+	0	1	0	0
pHV437	232853	233902	2	1049	+	0	0	0	2
pHV438	237010	238752	4	1742	-	0	1	0	2
pHV439	239082	242983	2	3901	-	5	6	0	0
pHV44	34050	34780	2	730	+	1	4	0	7
pHV440	246816	247418	2	602	-	2	5	1	4
pHV441	249905	250330	2	425	-	1	0	0	0
pHV442	253729	254132	2	403	+	0	1	1	2
pHV443	260858	261217	3	359	-	0	0	0	0
pHV444	268959	269635	3	676	-	0	0	0	0
pHV445	274083	277122	2	3039	+	1	0	0	0
pHV446	281180	283844	2	2664	-	2	2	1	0
pHV447	284563	285140	5	577	+	0	0	0	1
pHV448	289927	291826	2	1899	+	0	0	0	1
pHV449	291900	295042	3	3142	+	1	1	0	1
pHV45	40248	41372	2	1124	-	0	1	1	1
pHV450	297495	298723	2	1228	+	1	1	0	1
pHV451	303472	305785	2	2313	+	0	0	0	0
pHV452	305947	308231	2	2284	+	1	0	0	0
pHV453	324116	325835	2	1719	-	0	1	0	0
pHV454	326858	327691	3	833	-	0	1	1	2
pHV455	329325	329997	2	672	+	1	3	0	3
pHV456	332424	334635	3	2211	+	0	0	0	1
pHV457	338616	339682	2	1066	+	1	0	0	4
pHV458	346747	352444	4	5697	-	1	2	1	0
pHV459	356250	357006	2	756	-	0	0	0	0
pHV46	45861	46563	2	702	+	0	0	0	0
pHV460	356999	357564	2	565	-	0	0	0	0
pHV461	359324	360924	2	1600	-	1	1	0	0
pHV462	363010	364424	2	1414	+	3	2	1	1
pHV463	365782	370806	4	5024	+	2	6	0	0
pHV464	375206	376550	2	1344	+	0	7	0	7
pHV465	379569	380139	2	570	-	1	1	0	7
pHV466	380272	381015	4	743	+	1	1	0	0
pHV467	385336	391035	4	5699	-	3	18	1	7
pHV468	391212	397064	5	5852	+	4	6	1	2
pHV469	397167	401295	3	4128	+	5	5	1	0
pHV47	46903	48686	5	1783	+	0	0	0	0
pHV470	401900	402328	3	428	+	0	0	0	0
pHV471	402616	403319	3	703	+	2	2	0	0
pHV472	403385	404007	2	622	+	0	0	0	1
pHV473	404316	406098	3	1782	+	2	4	0	2
pHV474	410345	411435	3	1090	+	0	1	0	0
pHV475	413614	414760	2	1146	+	1	1	1	0
pHV476	422330	423086	2	756	+	0	1	1	2

pHV477	428467	434114	5	5647	-	6	2	1	13
pHV478	435957	438049	2	2092	-	5	1	1	3
pHV479	443575	445307	2	1732	-	1	1	1	7
pHV48	48734	50469	2	1735	+	1	0	0	0
pHV480	445533	446530	2	997	+	0	0	0	3
pHV481	447953	450711	2	2758	-	2	0	0	0
pHV482	451482	452332	2	850	-	0	1	0	0
pHV483	456082	457904	2	1822	-	2	9	0	6
pHV484	458433	461248	2	2815	+	4	4	1	2
pHV485	465053	466335	2	1282	+	1	2	0	0
pHV486	467011	468605	2	1594	-	2	0	0	0
pHV487	472030	476165	2	4135	+	5	2	1	0
pHV488	477582	479248	2	1666	+	0	0	0	1
pHV489	484636	488347	3	3711	+	0	0	0	0
pHV49	57789	61230	2	3441	-	0	4	1	4
pHV490	490874	492043	2	1169	+	0	6	0	1
pHV491	497237	503849	6	6612	-	0	0	0	0
pHV492	513415	516113	5	2698	+	0	0	0	1
pHV493	518259	521576	3	3317	-	1	0	0	0
pHV494	525323	528051	2	2728	+	0	0	0	1
pHV495	528138	529900	2	1762	+	0	0	0	1
pHV496	531423	533442	2	2019	+	0	0	0	0
pHV497	535259	536575	2	1316	-	0	0	0	0
pHV498	538172	543008	3	4836	-	1	0	0	0
pHV499	546030	546592	2	562	-	0	0	0	2

Supplementary Table 10. Operons that occur for all three replicates as outliers on top of the boxes in Supplemental Figure 7 (high TPM ratios) are listed. The table includes general information (rows with blue background) about the operon such as an ID, start and end coordinates, the number of internal TTS, the strand, the number of corresponding genes and the calculated ratios (in normal scale). For each operon, corresponding genes are then listed separately (rows with white background) with gene ID, start and end coordinates, strand, length and annotation.

**OperonID**	**start**	**end**	**numTTS**	**strand**	**numGenes**	**ratio1**	**ratio2**	**ratio3**
OperonID	geneID	start	end	strand	length	annotation		
**CHR70**	**393182**	**394581**	**2**	**+**	**3**	**8,56390112**	**7,43262821**	**12,405723**
CHR70	HVO_0442	393182	393352	+	170	conserved hypothetical protein		
CHR70	HVO_0443	393349	393960	+	611	UPF0215 family protein		
CHR70	HVO_0444	393985	394581	+	596	uracil-DNA glycosylase		
**CHR90**	**560896**	**564999**	**9**	**+**	**4**	**5,0374162**	**4,14919**	**8,40162162**
CHR90	HVO_0628	560896	562620	+	1724	ABC-type transport system periplasmic substrate-binding protein (probable substrate dipeptide/oligopeptide)		
CHR90	HVO_0629	562646	563665	+	1019	ABC-type transport system permease protein (probable substrate dipeptide/oligopeptide)		
CHR90	HVO_0630	563662	564597	+	935	ABC-type transport system permease protein (probable substrate dipeptide/oligopeptide)		
CHR90	HVO_0631	564604	564999	+	395	conserved hypothetical protein		
**CHR280**	**1747402**	**1745405**	**8**	**-**	**2**	**12,1143252**	**11,2187176**	**7,84578381**
CHR280	HVO_1895	1745405	1747063	-	1658	KEOPS complex subunit Kae1/Bud32		
CHR280	HVO_1896	1747097	1747402	-	305	30S ribosomal protein S24e		
**CHR369**	**2420295**	**2406014**	**66**	**+**	**25**	**15,5968751**	**18,6912142**	**6,33896205**
CHR369	HVO_2541	2406014	2407483	-	1469	protein translocase subunit SecY		
CHR369	HVO_2542	2407487	2407984	-	497	50S ribosomal protein L15		
CHR369	HVO_2543	2407981	2408445	-	464	50S ribosomal protein L30		
CHR369	HVO_2544	2408445	2409089	-	644	30S ribosomal protein S5		
CHR369	HVO_2545	2409086	2409640	-	554	50S ribosomal protein L18		
CHR369	HVO_2546	2409640	2410095	-	455	50S ribosomal protein L19e		
CHR369	HVO_2547	2410095	2410802	-	707	50S ribosomal protein L32e		
CHR369	HVO_2548	2410802	2411338	-	536	50S ribosomal protein L6		
CHR369	HVO_2549	2411343	2411735	-	392	30S ribosomal protein S8		
CHR369	HVO_2550	2411739	2411915	-	176	30S ribosomal protein S14		
CHR369	HVO_2551	2411912	2412430	-	518	50S ribosomal protein L5		
CHR369	HVO_2552	2412427	2413170	-	743	30S ribosomal protein S4e		

CHR369	HVO_2553	2413167	2413523	-	356	50S ribosomal protein L24		
CHR369	HVO_2554	2413525	2413923	-	398	50S ribosomal protein L14		
CHR369	HVO_2555	2413923	2414267	-	344	30S ribosomal protein S17		
CHR369	HVO_2556	2414258	2414689	-	431	ribonuclease P protein component 1		
CHR369	HVO_2557	2414689	2414901	-	212	50S ribosomal protein L29		
CHR369	HVO_2558	2414902	2415816	-	914	30S ribosomal protein S3		
CHR369	HVO_2559	2415816	2416277	-	461	50S ribosomal protein L22		
CHR369	HVO_2560	2416281	2416703	-	422	30S ribosomal protein S19		
CHR369	HVO_2561	2416706	2417431	-	725	50S ribosomal protein L2		
CHR369	HVO_2562	2417434	2417685	-	251	50S ribosomal protein L23		
CHR369	HVO_2563	2417682	2418428	-	746	50S ribosomal protein L4		
CHR369	HVO_2564	2418432	2419448	-	1016	50S ribosomal protein L3		
CHR369	HVO_2565	2419453	2420295	-	842	DUF171 family protein		
**CHR94**	**584621**	**583912**	**4**	**-**	**3**	**6,94407969**	**7,08547009**	**6,30854606**
CHR94	HVO_0652	583912	584181	-	269	KEOPS complex subunit Pcc1		
CHR94	HVO_0653	584183	584317	-	134	DNA-directed RNA polymerase subunit P		
CHR94	HVO_0654	584352	584621	-	269	50S ribosomal protein L43e		
**CHR322**	**2072992**	**2070415**	**11**	**-**	**3**	**4,44259932**	**3,77355278**	**5,86716301**
CHR322	HVO_2202	2070415	2071035	-	620	2-phospho-L-lactate guanylyltransferase		
CHR322	HVO_2203	2071042	2071836	-	794	conserved hypothetical protein		
CHR322	HVO_2204	2071805	2072992	-	1187	FtsZ family protein CetZ%2C type III		
**CHR248**	**1510012**	**1506162**	**1**	**-**	**4**	**4,88145802**	**4,37752107**	**4,67794641**
CHR248	HVO_1643	1506162	1506395	-	233	conserved hypothetical protein		
CHR248	HVO_1644	1506392	1506652	-	260	conserved hypothetical protein		
CHR248	HVO_1645	1506663	1509224	-	2561	cox-type terminal oxidase subunit I/III		
CHR248	HVO_1646	1509221	1510012	-	791	cox-type terminal oxidase subunit II		

Supplementary Table 11. Operons that occur for all three replicates as outliers below the boxes in Supplemental Figure 7 (low TPM ratios) are listed. The table includes general information (rows with blue background) about the operon such as an ID, start and end coordinates, the number of internal TTS, the strand, the number of corresponding genes and the calculated ratios (in normal scale). For each operon, corresponding genes are then listed separately (rows with white background) with gene ID, start and end coordinates, strand, length and annotation.

OperonID	start	end	numTTS	strand	numGenes	ratio1	ratio2	ratio3
OperonID	geneID	start	end	strand	length	annotation		
**pHV4113**	**129485**	**129841**	**2**	**+**	**2**	**0,00143893**	**0,00159007**	**0,00274036**
pHV4113	HVO_A0131s	129485	129672	+	187	NA		
pHV4113	HVO_A0131s2	129695	129841	+	146	NA		
**pHV4115**	**251268**	**250939**	**4**	**-**	**2**	**0,00792179**	**0,00498744**	**0,00424159**
pHV4115	HVO_A0249s	250939	251267	-	328	NA		
pHV4115	HVO_A0249s2	251252	251268	-	16	NA		
**CHR360**	**2348261**	**2344719**	**0**	**-**	**5**	**0,01477429**	**0,01630341**	**0,03220868**
CHR360	HVO_2475	2344719	2344907	-	188	30S ribosomal protein S17e		
CHR360	HVO_2476	2344932	2345621	-	689	DUF447 family protein		
CHR360	HVO_2477	2345618	2346478	-	860	triphosphoribosyl-dephospho-CoA synthase		
CHR360	HVO_2478	2346478	2347269	-	791	putative dihydropyrimidine dehydrogenase		
CHR360	HVO_2479	2347269	2348261	-	992	2-phospho-L-lactate transferase		
**CHR107**	**654895**	**653749**	**2**	**-**	**2**	**0,03573663**	**0,0344763**	**0,20032387**
CHR107	HVO_0731	653749	654309	-	560	conserved hypothetical protein		
CHR107	HVO_0732	654302	654895	-	593	ribonuclease H%2C type 1		
**CHR166**	**1000106**	**997547**	**8**	**-**	**4**	**0,07132162**	**0,06442633**	**0,02430074**
CHR166	HVO_1091	997547	998305	-	758	proteasome alpha subunit		
CHR166	HVO_1092	998312	998791	-	479	ribonuclease P protein component 2		
CHR166	HVO_1093	998801	999427	-	626	probable S-adenosylmethionine-dependent methyltransferase		
CHR166	HVO_1094	999411	1000106	-	695	ribonuclease P protein component 3		
**CHR393**	**2568180**	**2567777**	**1**	**-**	**2**	**0,07410786**	**0,06912966**	**0,19719058**
CHR393	HVO_2722	2567777	2567953	-	176	50S ribosomal protein L37e		
CHR393	HVO_2723	2567950	2568180	-	230	RNA-binding protein Lsm		
**CHR56**	**325966**	**327485**	**2**	**+**	**2**	**0,12807427**	**0,09923163**	**0,20049499**
CHR56	HVO_0357	325966	326535	+	569	ACT domain protein		

CHR56	HVO_0358	326532	327485	+	953	homoserine dehydrogenase		
**CHR158**	**949985**	**951300**	**1**	**+**	**2**	**0,14910386**	**0,14012671**	**0,2026799**
CHR158	HVO_1041	949985	950983	+	998	beta-lactamase domain protein		
CHR158	HVO_1042	951010	951300	+	290	DNA-directed RNA polymerase subunit L		
**CHR325**	**2082735**	**2082166**	**0**	**-**	**2**	**0,15483155**	**0,12106799**	**0,09546249**
CHR325	HVO_2218	2082166	2082510	-	344	conserved hypothetical protein		
CHR325	HVO_2219	2082520	2082735	-	215	hypothetical protein		
**CHR60**	**339210**	**338106**	**1**	**-**	**2**	**0,21546494**	**0,17159852**	**0,2281952**
CHR60	HVO_0379_A	338106	338621	-	515	conserved hypothetical protein		
CHR60	HVO_0379_B	338611	339210	-	599	hypothetical protein		

E. References

1. Tjaden, B. (2015) De novo assembly of bacterial transcriptomes from RNA-seq data. *Genome Biol.*, **16:1.**, 10.1186/s13059-13014-10572-13052.
2. Pfeiffer, F. and Oesterhelt, D. (2015) A manual curation strategy to improve genome annotation: application to a set of haloarchael genomes. *Life (Basel, Switzerland)*, **5**, 1427-1444.
3. Maier, L.K., Benz, J., Fischer, S., Alstetter, M., Jaschinski, K., Hilker, R., Becker, A., Allers, T., Soppa, J. and Marchfelder, A. (2015) Deletion of the Sm1 encoding motif in the lsm gene results in distinct changes in the transcriptome and enhanced swarming activity of Haloferax cells. *Biochimie*, **6**, 00058-00059.
4. Allers, T., Ngo, H.P., Mevarech, M. and Lloyd, R.G. (2004) Development of additional selectable markers for the halophilic archaeon Haloferax volcanii based on the leuB and trpA genes. *Appl Environ Microbiol*, **70**, 943-953.
5. Large, A., Stamme, C., Lange, C., Duan, Z., Allers, T., Soppa, J. and Lund, P.A. (2007) Characterization of a tightly controlled promoter of the halophilic archaeon Haloferax volcanii and its use in the analysis of the essential cct1 gene. *Mol Microbiol.*, **66**, 1092-1106. Epub 2007 Oct 1031.
6. Hartman, A.L., Norais, C.d., Badger, J.H., Delmas, S.p., Haldenby, S., Madupu, R., Robinson, J., Khouri, H., Ren, Q., Lowe, T.M. *et al.* (2010) The Complete Genome Sequence of Haloferax volcanii DS2, a Model Archaeon. *PLoS ONE*, **5**, e9605.
